# *Mycobacterium abscessus* glycopeptidolipids inhibit macrophage apoptosis and bacterial spreading by targeting mitochondrial cyclophilin D

**DOI:** 10.1038/cddis.2017.420

**Published:** 2017-08-24

**Authors:** Jake Whang, Yong Woo Back, Kang-In Lee, Nagatoshi Fujiwara, Seungwha Paik, Chul Hee Choi, Jeong-Kyu Park, Hwa-Jung Kim

**Affiliations:** 1 Department of Microbiology and Department of Medical Science, College of Medicine, Chungnam National University, Daejeon, South Korea; 2 Department of Food and Nutrition, Tezukayama University, Gakuenminami, Nara, Japan

## Abstract

*Mycobacterium abscessus* (MAB) is a species of nontuberculous mycobacteria (NTM) and a major causative pathogen of pulmonary diseases especially in patients with cystic fibrosis. MAB infection is notoriously difficult to treat because of its intrinsic or inducible resistance to most antibiotics. The rough (R) morphotype of MAB, lacking cell surface glycopeptidolipids (GPLs), is associated with more severe and persistent infection than the smooth (S) type; however, the mechanisms underlying the R type’s virulence and the relation with GPLs remain unclear. In this study, we found that R-type MAB is much more proapoptotic than the S type, as a result of GPL-mediated inhibition of macrophage apoptosis. Polar GPLs inhibited an apoptotic response (induced by proapoptotic stimuli) by suppressing ROS production and the cytochrome *c* release and by preserving mitochondrial transmembrane potential. Furthermore, GPLs were found to be targeted to mitochondria and interacted with cyclophilin D; their acetylation was essential for this interaction. Finally, GPLs inhibited the intracellular growth and bacterial spreading of R-type MAB among macrophages via apoptosis inhibition. These findings suggest that GPLs limit MAB virulence by inhibiting apoptosis and the spread of bacteria and therefore provide a novel insight into the mechanism underlying virulence of MAB.

*Mycobacterium abscessus* (MAB) is a rapidly growing nontuberculous mycobacterium (NTM) and is a common causative agent of lung diseases especially in cystic fibrosis patients.^1, 2^ MAB infection is notoriously difficult to treat because of its intrinsic or inducible resistance to most antibiotics.^3, 4^ Although the isolation rate of NTM species such as *M. avium* (MAV), MAB, and *M. kansasii* in clinical samples is rapidly increasing, little is known about their virulence factors and their pathogenicity at the cellular level.

MAB has two morphological forms (morphotypes): smooth (S) and rough (R). The major difference between these types is the loss of surface-associated glycopeptidolipids (GPLs) in the R type.^5, 6^ S-type MAB appears to be predominant in the environment and is mostly associated with an initial infection, whereas the R morphotype is associated with more severe and persistent infection.^7, 8^ Nonetheless, it is still unclear why the R type is more virulent than the S type.

GPLs are produced by most NTM species^9, 10, 11^ and share a common diglycosylated N-linked long fatty acyl chain linked to a tetrapeptide-amino-alcohol core but show differences in modifications consisting of attached rhamnose and 6-deoxytalose. The GPL core forms apolar or nonspecific GPLs (nsGPLs) that are produced by all NTM species, whereas polar, serotype-specific GPLs (ssGPLs) vary depending on the strain.^12, 13^ MAB produces unique polar GPLs that are diglycosylated on alaninol and di-*O*-acetylated on 6-deoxytalose.^12, 13^ MAB GPLs mask underlying bioactive cell wall lipids, thereby delaying the immunity activation during the initial colonization stages,^14^ indicating their role as a virulence factor. In addition, GPLs facilitate MAB colonization by preventing Toll-like receptor signaling in airway epithelial cells.^15, 16^ Nevertheless, the precise pathogenic role of GPLs during MAB infection remains unclear.

Macrophages are the primary target and an important reservoir of mycobacteria in lungs, as indicated by the fact that virulent mycobacteria can survive and replicate within macrophages. Apoptotic cell death is regarded as an innate intracellular response designed to limit multiplication of intracellular pathogens.^17, 18^ A high intracellular burden of virulent *M. tuberculosis* induces apoptosis with rapid progression to necrosis as a mode of mycobacterial escape.^19^ It was also suggested that *M. avium* uses apoptotic macrophages as a tool for cell-to-cell spreading in the host.^20^ We also have reported that highly virulent clinical MAB strains induce significantly more cell death than nonvirulent strains.^21^ On the other hand, the pathogenic role of cell death in a virulent MAB infection remains largely unknown. Recently, a study on a zebrafish infection model indicated that R-type MAB induces more macrophage apoptosis than the S type and the virulence correlates with a loss of GPL production and massive production of a serpentine cord.^22^ In the present study, we examined the precise role of GPLs in virulence and why the R type is more virulent than S type. Here, we used extracted GPLs, studied their targeting to mitochondria, and their function in macrophage apoptosis.

## Results

### R-type MAB induces macrophage apoptosis significantly more than the S type

**Figure 1:**
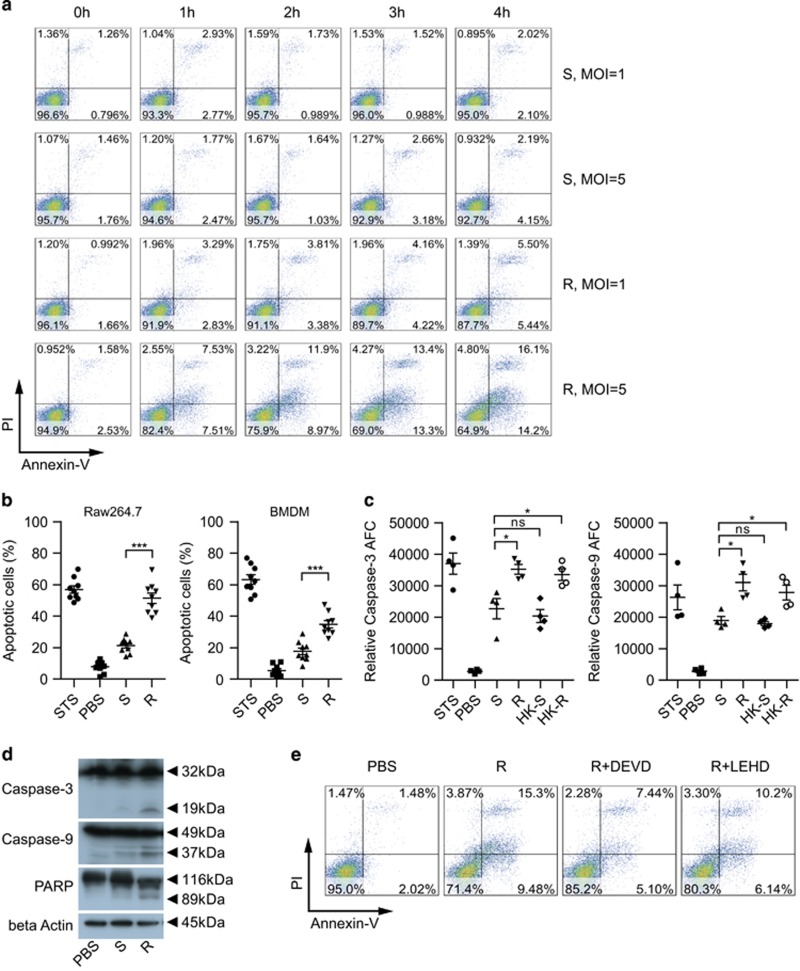
R-type MAB induces significantly more macrophage apoptosis than the S type. (**a**) RAW 264.7 cells were infected with R- or S-type MAB for 1 to 4 h at MOIs of 1 and 5 without washing to remove extracellular bacteria. The apoptotic responses were analyzed by flow cytometry using annexin V/PI staining. (**b**) RAW 264.7 cells and BMDMs infected with R- or S-type MAB (MOI=5) for 2 h were washed three times with PBS, and apoptotic cells were detected by a TUNEL assay at 6 h after the final washing. Treatment with staurosporine (STS; 100 nM) served as a positive control for 8 h. Data are shown as mean±S.E.M. from three independent experiments (*n*=3 per group). (**c**) Caspase 3 and caspase 9 activities of RAW 264.7 infected with live or heat-killed R-type (HK-R) or S-type (HK-S) MAB (MOI=5) for 4 h were determined using a caspase activity assay kit. Treatment with (100 nM) served as a positive control for 6 h. Results are shown as mean±S.D. (*n*=4). One-way or two-way ANOVA was used for group comparisons. *P<0.05, ***P<0.001; NS, not significant in (**b**) and (**c**). (**d**) Total lysates from RAW 264.7 cells MAB infected for 6 h were immunoblotted against caspases 3 and 9 and PARP. (**e**) RAW 264.7 cells were infected with R-type MAB (MOI=5) and treated with each apoptosis inhibitor at 50 *μ*M: Z-DEVD-Fmk for caspase 3 (DEVD) and Z-LEHD-Fmk for caspase 9 (LEHD). The apoptotic population was identified by FACS analysis using annexin V/PI staining

To elucidate the relation between apoptosis and MAB virulence, we compared the apoptotic response of macrophages infected with either S or R morphotype. In the first group, RAW 264.7 cells were infected with MAB at multiplicity of infections (MOIs) of 1 and 5, and then their cell death pattern was analyzed by fluorescence-activated cell sorting (FACS) with annexin V/propidium iodide (PI) staining from 1 to 4 h with 1 h intervals. The R type induced apoptosis in a time- and dose-dependent manner: significantly more apoptosis than the S type (Figure 1a). The S morphotype did not induce substantial apoptosis at MOI of 1 and induced prominent apoptosis only at MOI of 5 at 4 h after infection. The cell death progressed rapidly in R-type-infected RAW 264.7 cells. The difference in the cell death induced by R and S types was not due to a difference in bacterial clumping as indicated by acid fast bacilli (AFB) staining of both bacterial types prepared for infection (Supplementary Figure 1a), and there was no difference in the macrophage infection rate between the two types (Supplementary Figure 1b). Next, MAB-induced apoptosis was confirmed in RAW 264.7 cells (by a TUNNEL assay) that were infected with either of the types of MAB for 2 h and washed three times with PBS. At 6 h after final washing, the R type induced significantly more apoptosis in RAW 264.7 cells than the S type (Figure 1b). Similar results were observed in primary cells: bone marrow-derived macrophages (BMDMs; Figure 1b). To rule out an effect of extracellularly growing bacteria, BMDMs that were MAB infected for 2 h were treated with kanamycin to kill extracellular bacteria, were washed three times with PBS, and then were cultured in a medium containing kanamycin. At 48 h post infection, the R type induced significantly more apoptosis as compared with the S type (Supplementary Figure 2a).

We then tested whether MAB induced apoptosis in a caspase-dependent manner. Western blot analysis revealed the presence of cleaved forms of caspase 3, caspase 9, and PARP in RAW 264.7 cells infected with the R type (Figure 1d). Caspase 3 and 9 activities were significantly higher in R-type-infected than in S-type-infected macrophages (Figure 1c). R-type-induced apoptosis was inhibited by caspase 3- and 9-specific inhibitors (Figure 1e). Overall, these results indicated that the R type induces robust caspase-dependent apoptosis in macrophages when compared with the S type’s effects.

### Polar GPLs of the S type inhibit intrinsic apoptosis of macrophages

**Figure 2:**
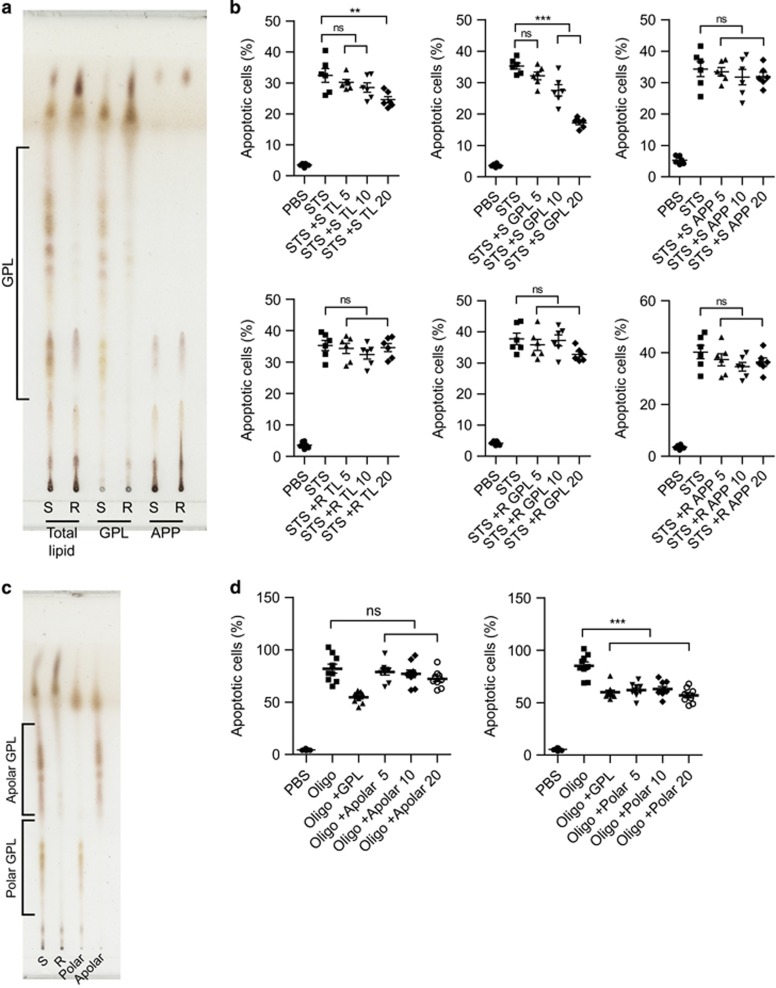
Polar GPLs from the S type inhibit intrinsic apoptosis of macrophages. (**a**) The purified GPLs and the acetone-precipitated pellet (APP) from total lipids extracted from R and S types were analyzed by thin-layer chromatography (TLC), using chloroform/methanol (9 : 1, v/v) as a mobile phase. The resolved GPLs are indicated with a bracket. (**b**) Effects of pretreatment with total lipid extracts, purified GPLs, or APP from S-type MAB (5, 10, or 20 *μ*g/ml) for 1 h on STS (100 nM)-induced death of RAW 264.7 cells. (**c**) The GPLs purified by TLC from S-type MAB (S-GPL) were then separated into polar and apolar fractions. The resolved polar and apolar GPLs are indicated with a bracket. (**d**) RAW 264.7 cells were pretreated with apolar or polar GPLs of S-type MAB for 1 h and then stimulated with oligomycin (Oligo, 5 *μ*g/ml). (**b** and **d**) After 24 h, the apoptotic responses of RAW 264.7 cells were analyzed by FACS with annexin V/PI staining. The population of apoptotic cells was calculated from data in the lower and upper right quadrants (Q2, Q4; Annexin V+, PI±). Data are shown as mean±S.E.M. from three experiments (*n*=3 per group). **P<0.01, ***P<0.001 (one-way ANOVA); NS, not significant

Several apoptosis-regulating proteins and glycolipids of *M. tuberculosis* have been reported.^23, 24^ In an assay designed to identify the apoptosis-inducing component of MAB, we found that heat killing of the bacteria did not result in a significant decrease in R-type-induced macrophage apoptosis (Supplementary Figure 2b) or caspase activities (Figure 1c). In addition, there was no difference in cell survival effects between culture filtrate proteins from R- and S-type MAB (Supplementary Figure 3). These results suggested that lipids rather than proteins are the apoptosis-modulating factors of MAB. Therefore, we hypothesized that the differential apoptotic response induced by the two morphotypes may be due to a difference in the amount of cell wall lipids. As expected, a major difference in total cell wall lipids between S and R types was observed in thin-layer chromatography (TLC) analysis (Figure 2a) and two-dimensional TLC (Supplementary Figure 4) in the GPL region. Total lipids and purified GPLs from R-type MAB showed a few spots in the GPL region. The acetone-precipitated pellet (APP), where GPLs were removed, yielded the same pattern in both morphotypes. To quantitatively compare the GPL amounts between both types, we purified mycolic acid, total lipid extract, GPL, and APP from the bacteria (Supplementary Figure 5). The amount of the mycolic acid in both types was same, but the GPLs in S type were 3.1-fold higher than R type when the GPLs were purified from 10 mg of the total lipid extracts of each type (Supplementary Table 1). Some spots in the GPL region on TLC were analyzed by matrix-assisted laser desorption ionization time-of-flight mass spectrometry (MALDI-TOF/MS). We confirmed that the small spots of APP that appeared in the GPL region were not lipids of the GPL fraction (Supplementary Figure 6).

Next, we analyzed the potential participation of GPLs in the inhibition of macrophage apoptosis. GPLs did not induce any apoptosis (data not shown). Total lipids and purified GPLs from the S type, but not APP or total lipids of the R type, inhibited staurosporine (STS)-induced apoptosis in a dose-dependent manner (Figure 2b and Supplementary Figure 7). Furthermore, GPLs inhibited apoptosis induced by oligomycin or H_2_O_2_ but did not inhibit apoptosis induced by TNF-*α* plus cycloheximide (Supplementary Figure 7). These results suggested that GPLs modulate the intrinsic apoptotic response of macrophages.

To further elucidate the compounds participating in the modulation of apoptosis, the GPLs were fractionated into polar and apolar GPLs (Figure 2c). Polar GPLs, but not apolar GPLs, significantly inhibited oligomycin-induced apoptosis of macrophages (Figure 2d). In fact, R-type GPLs consisted of only a small amount of apolar GPLs that were not involved in the inhibition of apoptosis. GPLs deacetylated by alkaline lysis (dGPLs) showed a loss of their antiapoptotic effects (Supplementary Figure 8). These results suggested that polar GPLs are mainly responsible for inhibiting macrophage apoptosis, and their acetylation is necessary for this activity.

### GPLs inhibit ROS production and a cytochrome *c* release from mitochondria and preserve mitochondrial transmembrane potential (ΔΨm)

**Figure 3:**
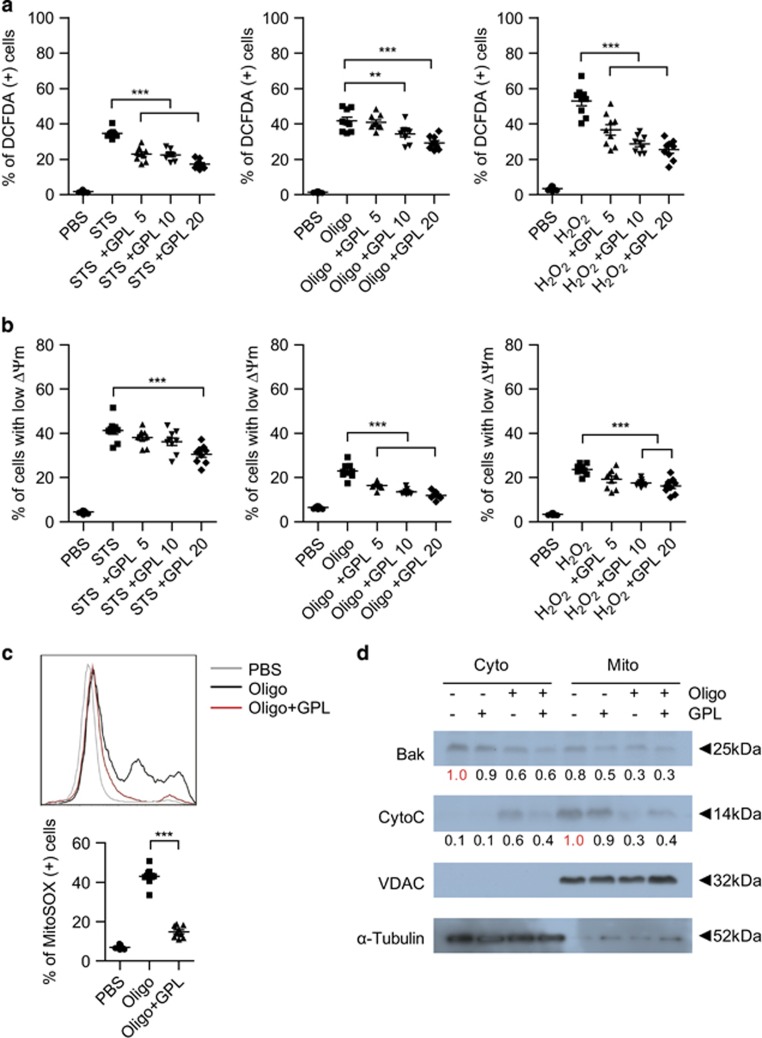
GPLs inhibit ROS production and cytochrome *c* release from mitochondria and preserve mitochondrial transmembrane potential (ΔΨm). Cellular ROS production (**a**) and ΔΨm (**b**) under the influence of staurosporine (STS, 10 nM), H_2_O_2_ (0.5 mM), or oligomycin (Oligo, 5 *μ*g/ml) in RAW 264.7 cells for 24 h with or without pretreatment with GPLs (5, 10, or 20 *μ*g/ml) were measured by flow cytometry using dichlorofluorescein diacetate (DCFDA) and DiOC_6_, respectively. (**c**) In RAW 264.7 cells pretreated with GPLs (20 *μ*g/ml), oligomycin-induced mitochondrial ROS production was measured by flow cytometry using the MitoSOX dye. Representative data from three experiments are shown. The data (**a**–**c**) are presented as mean±S.E.M. from three experiments (*n*=3 per experiment). **P<0.01, ***P<0.001 (one-way ANOVA). (**d**) Pretreatment with GPLs for 1 h inhibited an oligomycin-induced cytochrome *c* (CytoC) release in RAW 264.7 cells. Mitochondrial and cytosolic fractions were prepared and subjected to western blot analysis and probed with antibodies against Bak and CytoC. VDAC and *α*-tubulin served as markers of the mitochondrial and cytosolic fraction, respectively. The relative intensities of Bak and CytoC were measured by densitometry and normalized to the band intensity of each negative control of the cytosolic and mitochondrial fraction

It is well known that enhanced production of reactive oxygen species (ROS) can cause apoptosis. We examined the involvement of cellular ROS in GPL-mediated inhibition of apoptosis in RAW 264.7 cells. GPLs significantly inhibited STS-, H_2_O_2_-, or oligomycin-induced cellular ROS production in a dose-dependent manner (Figure 3a). Next, we evaluated the effects of GPLs on ΔΨ_m_ by flow cytometric analysis using 3,3′-dihexyloxacarbocyanine (DiOC_6_) staining. Pretreatment of RAW 264.7 cells with GPLs prevented disruption of ΔΨ_m_ by oligomycin, STS, or H_2_O_2_ (Figure 3b). Mitochondria play a central role in the response to apoptotic stimuli and are the major source of ROS in the cell. In macrophages, GPLs dramatically inhibited mitochondrial ROS production induced by oligomycin, a typical apoptosis inducer affecting mitochondria (Figure 3c). Furthermore, GPLs downregulated translocation of cytochrome *c* from mitochondria to the cytosol (Figure 3d) but did not significantly affect Bak translocation. Collectively, these results indicated that GPLs modulate apoptosis via their interaction with mitochondria.

### GPLs are targeted to mitochondria

**Figure 4:**
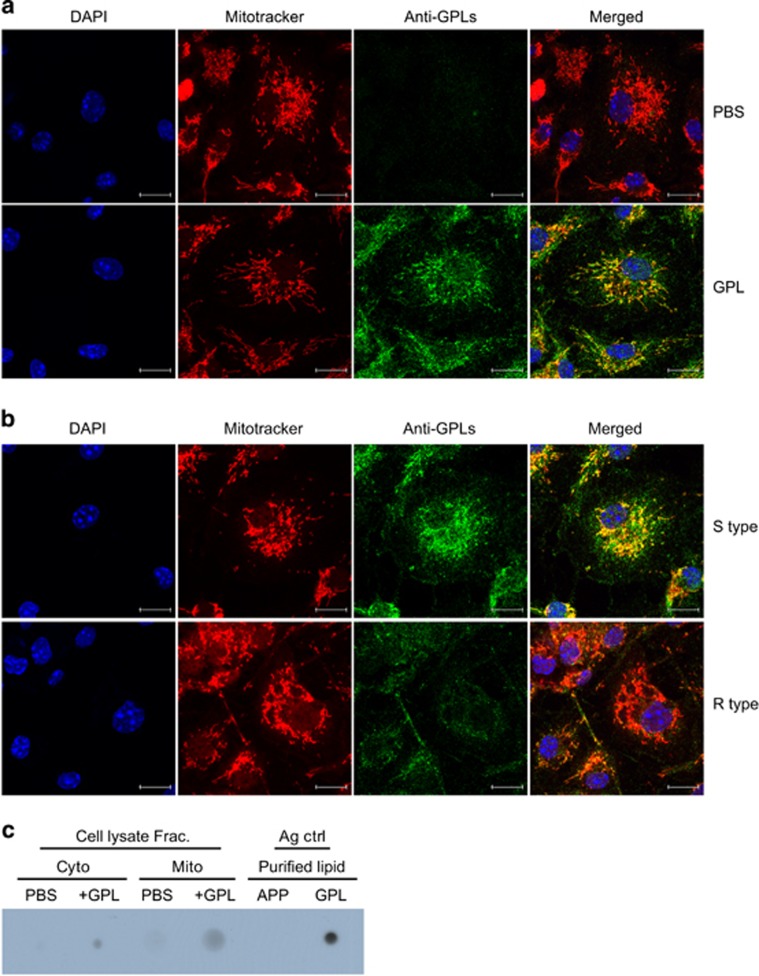
GPLs are targeted to mitochondria. (**a** and **b**) A confocal microscopy image of colocalization of GPLs in BMDMs treated with GPLs or left untreated for 4 h or infected with S- or R-type MAB (MOI=5) for 4 h. The cells were double stained with MitoTracker (red) and an anti-GPL antibody (green) followed by incubation with an Alexa 488-conjugated secondary antibody. DAPI (blue) was used to counterstain nuclei. The yellow signal in the merged images indicates colocalization. (**c**) Mitochondrial (Mito) and cytosolic (Cyto) fractions were separated from cells treated with GPLs and were analyzed by dot blotting with specific antibodies against GPLs. Purified GPLs and APP were dot-blotted as positive and negative controls (Ag ctrl, antigen control)

Next, we determined where GPLs are localized in GPL-treated cells. GPLs were found to be colocalized with mitochondria in GPL-treated cells as evidenced by confocal microscopy (Figure 4a). We also found that a considerable proportion of GPLs in S-type-infected macrophages colocalized with mitochondria (Figure 4b). Only a few anti-GPL antibody-immunostained mitochondria were observed in R-type-infected cells when compared with S-type-infected cells, because of the low GPL content of the R-type MAB. Given that GPLs were not separated by sodium dodecyl sulfate polyacrylamide gel electrophoresis (SDS-PAGE), their location in the subcellular fractions of GPL-treated cells was analyzed by dot blotting. As expected, the anti-GPL antibody reacted with purified GPL spots used as a positive control and did not react with the APP spots serving as a negative control (Figure 4c). A positive reaction with the anti-GPL antibody was detected in both the cytosolic and mitochondrial fractions of the cells treated with GPLs. These findings revealed that GPLs are efficiently transported to mitochondria in S-type MAB-infected macrophages.

### GPLs interact with cyclophilin D, a component of the MPT pore

**Figure 5:**
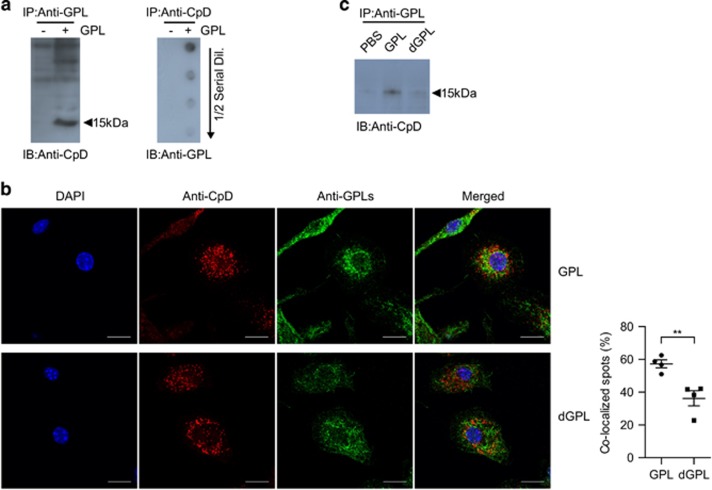
GPLs interact with cyclophilin D (CpD), and their acetylation is important for this interaction. (**a**) Cell lysates of RAW 264.7 cells treated with GPL (20 *μ*g/ml) or the culture medium alone for 2 h were subjected to immunoprecipitation with the anti-GPL antibody and were analyzed by western blotting with an anti-CpD antibody (left panel). The GPLs immunoprecipitated by the anti-CpD antibody were serially diluted on a half-log scale, spotted onto the membrane, and incubated with the anti-GPL antibody (right panel). (**b**) BMDMs were treated with GPLs or dGPLs for 2 h, fixed, and stained with antibodies against GPL and CpD. Colocalization of GPLs and dGPL with CpD was confirmed as yellow dots in the merged images. The colocalized GPLs were quantified by means of the LAS-X software from Leica Corporation (Leica, Wetzlar, Germany). Significant colocalization is presented as mean±S.D. (*n*=4), **P<0.01 (one-way ANOVA). (**c**) The cell lysates treated with GPLs or deacetylated GPLs (dGPL) were subjected to immunoprecipitation with an anti-GPL antibody and were analyzed by western blotting with the anti-CpD antibody

It is important to identify the host molecules that physically and functionally interact with GPLs for elucidation of their apoptosis-modulating mechanism. Therefore, we tested whether GPLs interact with pro- or anti-apoptotic molecules and mitochondrial permeability transition (MPT) pore components. Immunoprecipitation with the anti-GPL antibody revealed that GPLs bind to cyclophilin D (Figure 5a), a modulator of MPT pore opening.^25^ GPLs immunoprecipitated with the anti-cyclophilin D antibody were subjected to dot blotting with serial dilution that confirmed that cyclophilin D is coimmunoprecipitated with GPLs (Figure 5a). Furthermore, confocal microscopic analysis of GPL-treated cells revealed that GPLs colocalized with cyclophilin D (Figure 5b). Although dGPLs were colocalized with mitochondria, their colocalization rate was significantly lower than that of GPLs (Figure 5b). In contrast, dGPLs did not interact with cyclophilin D because the amount of cyclophilin D that was immunoprecipitated with dGPLs was much smaller than that immunoprecipitated with GPLs (Figure 5c). These results indicated that GPLs interact with the MPT pore component cyclophilin D, thus stabilizing mitochondrial membrane transition, and their acetylation may be crucial for the interaction with cyclophilin D.

### GPLs inhibit the apoptotic response induced by R-type MAB

**Figure 6:**
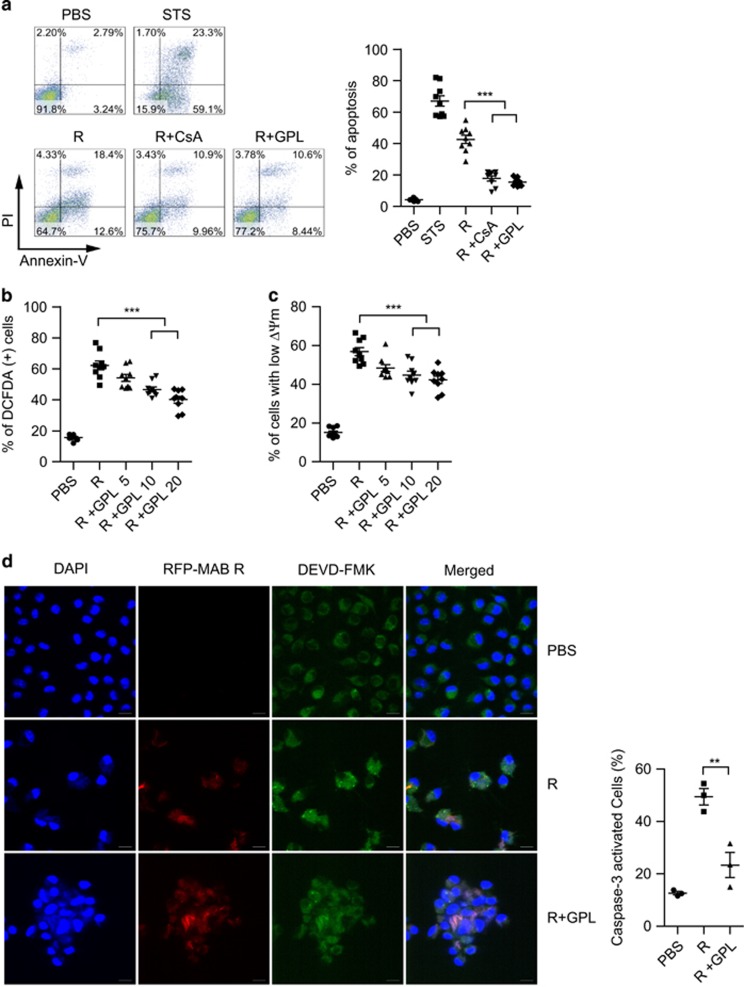
GPLs inhibit the apoptotic responses induced by R-type MAB. RAW 264.7 cells were pretreated with cyclosporin A (CsA, 10 *μ*g/ml) or GPLs (20 *μ*g/ml) for 2 h and incubated with R-type MAB (MOI=5) for 4 h. Staurosporine treatment (STS, 100 nM) served as a control. (**a**) Antiapoptotic effects of GPLs and CsA were measured by FACS with annexin V/PI staining. Data are shown as mean±S.E.M. from three independent experiments (*n*=3 per experiment). ***P<0.001. ROS (**b**) and ΔΨm (**c**) were quantified by FACS using DCFDA and DiOC_6_ staining, respectively. Data are presented as mean±S.E.M. from three independent experiments (*n*=3 per experiment), and one-way ANOVA was used for group comparisons. **P<0.01, ***P<0.001. (**d**) BMDMs pretreated with GPLs or PBS were infected with R-type MAB (MOI=5) for 4 h. Activated caspases were detected with DEVD-FMK that bind specifically to activated caspase 3, respectively. Representative confocal images of RFP-R-type MAB (red), DEVD-FMK staining (green), and DAPI staining of nuclei (blue) are shown. The significant activated cell ratios are presented as mean±S.D. (*n*=3), **P<0.01 (one-way ANOVA)

To confirm the roles of GPLs during infection, we determined whether GPLs could inhibit the R-type-induced apoptotic response in macrophages. The GPLs indeed inhibited R-type-induced macrophage apoptosis (Figure 6a). It is well known that cyclosporin A specifically binds cyclophilin D, leading to inhibition of MPT and apparently blocking apoptosis.^25, 26^ Just as GPLs, cyclosporin A inhibited R-type-induced macrophage apoptosis (Figure 6a). ROS production induced by the R-type MAB was also significantly inhibited after pretreatment with GPLs (Figure 6b). Moreover, GPLs prevented ΔΨm disruption induced by R-type MAB (Figure 6c). R-type MAB induced activation of caspases 3 and 9 in macrophages (Figure 1a). We measured caspase activity in macrophages infected with red fluorescent protein (RFP)-expressing R-type MAB. Activated caspase 3 specifically bound to the green-fluorophore-conjugated inhibitor and showed bright spots in a confocal image (Figure 6d). These spots were significantly reduced by cotreatment with GPLs. These results suggested that the stronger apoptotic response induced by R-type MAB in macrophages when compared with the S type may be caused by the lack of GPLs.

### GPLs inhibited the intracellular growth and the spreading of R-type MAB via apoptosis inhibition

**Figure 7:**
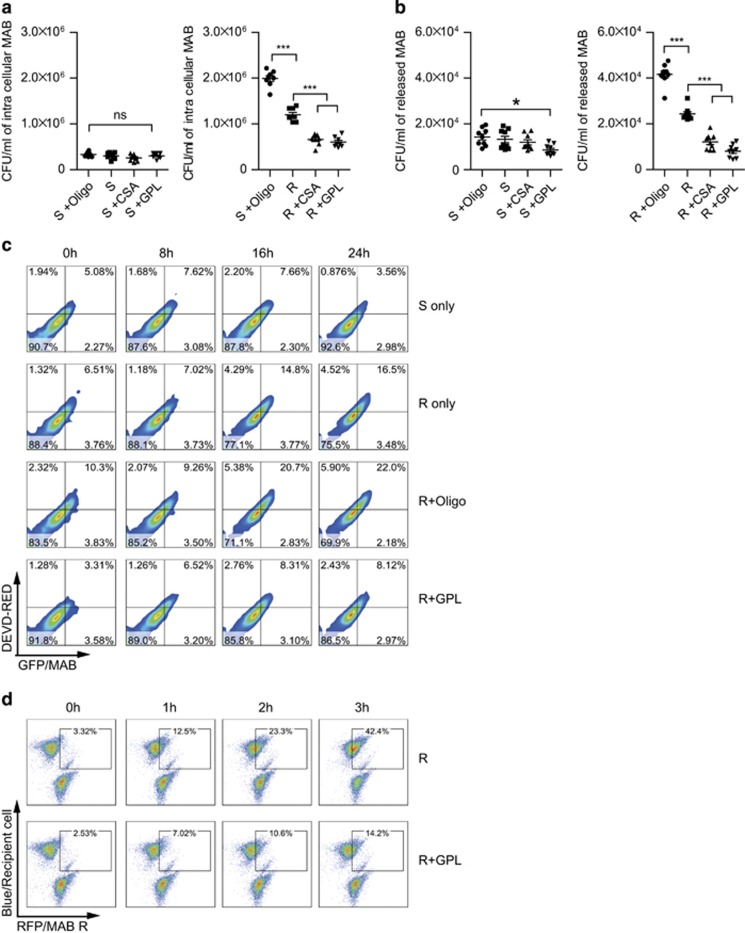
GPLs inhibit intracellular growth and the spread of R-type MAB among host cells via apoptosis inhibition. (**a** and **b**) BMDMs pretreated with PBS, GPLs (10 *μ*g/ml), cyclosporin A (CsA, 10 *μ*g/ml), or oligomycin (oligo, 5 *μ*g/ml) for 2 h were infected with S-type or R-type MAB (MOI=5) for 2 h, incubated for additional 2 h with kanamycin, and washed three times with PBS. At 24 h, the intracellular and extracellular MAB growth was measured by plating of the cell lysates and culture supernatants, respectively, on 7H9 agar. The bacterial CFU data are shown as mean±S.E.M. from three experiments (*n*=3 per experiment). *P<0.05, ***P<0.001; ns, not significant. (**c**) BMDMs infected with GFP-expressing MAB for 2 h were mixed with uninfected BMDMs. For 24 h at 8 h intervals, total BMDMs were analyzed by FACS using DEVD-red, a dye specific for activated caspase 3. (**d**) The bacterial spreading assay and FACS analysis were performed on RAW 264.7 cells. After the infection with RFP-expressing R-type MAB (MOI=5) for 2 h, the infected cells were collected and mixed with uninfected RAW 264.7 cells stained with a blue dye. Every hour for 3 h, the quantity of bacteria that entered the recipient cells was analyzed by FACS

It is well known that the R type is more virulent than the S type in terms of MAB infection, as proven in studies on survival of MAB-infected mice and bacterial growth in lungs.^8^ At the cellular level, the intracellular survival rate of the mycobacteria in macrophages allows for indirect assessment of their virulence. Therefore, we analyzed the relation between GPL-mediated inhibition of apoptosis and MAB pathogenicity by determining the effect of GPLs on intracellular survival of the R and S types in macrophages. BMDMs were infected with MAB for 2 h, incubated for additional 2 h with kanamycin to kill extracellular bacteria, washed three times with PBS, and then intracellular growth was determined. At 24 h after infection, the growth of the R type within macrophages was significantly greater when compared with that of the S type (Figure 7a). Moreover, pretreatment with GPLs or cyclosporin A significantly suppressed both the intracellular growth and extracellular release of the R type (Figures 7a and b). In contrast, pretreatment with oligomycin significantly enhanced both intracellular and extracellular growth of the R type. In contrast, the growth of the S type was not affected by GPLs or oligomycin pretreatment.

It is reported that *M. avium* uses apoptotic macrophages as a tool for spreading in the host.^20^ Therefore, we analyzed the correlation between apoptosis and the spread of bacteria. In the first assay, BMDMs infected with GFP-expressing R- or S-type MAB for 2 h were added with uninfected BMDMs. All the BMDMs were analyzed by FACS using DVED-RED staining every 8 h. The number of double-positive cells (that were infected with GFP MAB and contained activated caspase 3 simultaneously) significantly increased among R-type-infected BMDMs in a time-dependent manner as compared with S-type-infected cells (Figure 7c), indicating an increase in the infected cell numbers. The proportion of the double-positive cells induced by the R type was increased and decreased by pretreatment with oligomycin and GPLs, respectively.

To confirm the effect of GPLs on the spread of bacteria, RAW 264.7 cells infected with RFP-expressing R-type MAB for 2 h were added with uninfected cells stained with a blue dye. FACS analysis revealed that the proportion of R-type-infected cells among blue-stained RAW 264.7 cells increased in a time-dependent manner but was significantly inhibited by GPL pretreatment (Figure 7d). Collectively, these data suggest that apoptosis induced by R-type MAB may contribute to the spread of bacteria.

## Discussion

We have previously reported that highly virulent clinical MAB strains induce significantly more cell death than nonvirulent strains.^21^ It was recently reported that MAB of the S type is less proapoptotic than the R type.^27^ In the present study, we found that GPLs treatment inhibit the apoptotic response via mitochondrial targeting, thereby suppressing the growth of R-type MAB, resulting in inhibition of spreading of MAB among host cells. The data suggest that the low virulence of S-type MAB is associated with the apoptosis-inhibitory activity of the GPLs that are located on the outermost surface.

A variety of key events of cell death take place in mitochondria, including the cytochrome *c* release, loss of ΔΨ_m_, ROS production, and participation of apoptosis-regulating factors.^28^ We found that GPL-mediated inhibition of apoptosis resulted from downregulation of mitochondrial ROS (and of the release of cytochrome *c*) and was caused by protection from a ΔΨ_m_ loss. Moreover, GPLs were found to be localized to the mitochondrial compartment in macrophages infected with MAB as well as in macrophages treated with GPLs, although the mechanism of GPL internalization by the host cells remains unknown. Mitochondrial damage has been suggested to play a critical role in the outcome of infection of macrophages with *M. tuberculosis*.^29^ We recently demonstrated that the *M. tuberculosis* HBHA protein is targeted to the mitochondria and induces mitochondria-dependent apoptosis in macrophages.^30^ In the present study, MAB GPLs inhibited macrophage apoptosis via inhibition of mitochondrial damage induced by apoptotic stimuli. *Neisseria meningitides* porin B interacts with mitochondrial porin VDAC, resulting in enhanced cell survival.^31^ Of note, our findings showed that MAB GPLs, just as cyclosporin A, interact with cyclophilin D, leading to inhibition of MPT and of macrophage apoptosis. Inhibitors of MPT pore opening such as cyclosporin A appear to block cell death.^25, 26^ In our experimental model, MAB GPLs prevented disruption of ΔΨ_m_ induced by R-type MAB and by proapoptotic stimuli. MAB GPLs and cyclosporin A equally inhibited MAB-induced macrophage apoptosis. These results suggest that MAB GPLs interfere with the regulatory role of cyclophilin D in MPT pore opening, although further research into the underlying mechanisms is needed.

It is known that NTM species produce unique polar GPLs. MAB produces specific polar GPLs that are diglycosylated and diacetylated on the core GPL structure. In the present study, the polar GPL region was found to be involved in the inhibition of macrophage apoptosis. It is reported that proper acetylation and methylation of *M. avium* GPLs are required for TLR signaling and activation of the innate immune response.^32^ In the present study, acetylation of MAB GPLs was found to be necessary for both the inhibitory activity and interaction with cyclophilin D because dGPLs did not bind to cyclophilin D and did not have any antiapoptotic effect.

Macrophage death is an important characteristic of host–mycobacteria interactions. Therefore, the properties of mycobacteria that modulate apoptosis have been extensively studied. Most reports have indicated that the induction of apoptosis by *M. tuberculosis* and *M. avium* is inversely proportional to bacterial virulence.^33^ Nevertheless, the function of apoptosis in infection is still a subject of debate.^34^ RD1-deficient *M. tuberculosis* cannot trigger apoptosis and shows an impaired ability to colonize new uninfected cells, suggesting that apoptosis promotes cell-to-cell spread of bacteria.^34, 35, 36^ The importance of ESX-1-dependent apoptosis in the spread of *M. marinum* among host cells has been shown in the zebrafish model.^37^ In addition, apoptosis appears to act as a virulence mechanism for *M. leprae*.^38, 39^ Therefore, induction of apoptosis can apparently serve different functions according to the mycobacterial strain and its infection course. In the present study, inhibition of apoptosis by GPLs suppressed the growth of the virulent R type; however, induction of apoptosis by oligomycin enhanced bacterial growth. Furthermore, the increased apoptosis was found to be associated with spreading of the bacteria among host cells. Recently, a significant increase in the proportion of apoptotic macrophages was demonstrated in R-type-infected zebrafish as compared with S-type-infected macrophages.^22^ These authors also suggested that macrophage apoptosis is a key event for the release of extracellular MAB. In the present study, extracellular R-type MAB growth rapidly increased starting at 24 h after infection when compared with S-type MAB. Extracellular growth of R-type MAB was enhanced by treatment with an apoptosis inducer and was suppressed by GPLs or cyclosporin A. In agreement with these results, cyclosporin A suppressed intracellular growth of *M. tuberculosis* in macrophages by inhibiting necrosis of the infected macrophages.^40^ Nonetheless, in our experimental model, GPLs and cyclosporin A appeared to attenuate the increase in the number of annexin V-positive macrophages induced by R-type MAB and inhibited both extracellular and intracellular growth of the bacteria. Therefore, this discrepancy may due to a difference in the growth rate and apoptotic response between *M. tuberculosis* and MAB. Further research on the various characteristics of cell death modulated by GPLs and cyclosporine A is needed. Taken together, our findings reveal that R-type-induced apoptosis contributes to the spread of MAB and provides novel insights into the cellular pathogenic mechanism of R-type MAB and why it is more virulent than the S type. In line with our results, another study indicated that *M. avium* uses apoptotic macrophages as a vector for spreading.^20^ However, further studies are needed to investigate whether GPLs can also inhibit alveolar macrophages apoptosis *in vivo* that can suppress the spread and growth of MAB R type and attenuate the virulence.

## Materials and Methods

### Animal experiments

Male inbred C57BL/6 mice (5–6 weeks old) were used for preparation of the BMDMs. All the animal experiments were approved by the institutional research and ethics committee at Chungnam National University (permission number: CNU-00405). All the animal procedures were performed in accordance with the guidelines of the Korean Food and Drug Administration.

### Bacterial culture and GPL purification

*M. abscessus* ATCC19977 and the isogenic R type were grown to mid-log phase in the Middlebrook 7H9 medium containing OADC (BD, Franklin Lakes, NJ, USA), were washed three times, aliquoted, and frozen at −80 °C until use. Bacterial total lipids and GPLs were purified, solubilized, and confirmed as described previously.^41, 42, 43^ Total lipids were extracted from *M. abscessus* with a chloroform/methanol mixture (2 : 1, v/v) by ultrasonication for 20 min and were phase-separated with 5% distilled water by centrifugation. GPLs were purified from total lipid extracts by acetone precipitation. The purified lipids were separated by TLC (Millipore, Billerica, MA, USA) in chloroform/methanol (9 : 1, v/v) and detected by spraying with 20% H_2_SO_4_ and heating at 200 °C for 15 min. Two-dimensional TLC and visualization of phospholipids and amine groups were performed by means of ninhydrin and Dittmer’s solutions. Purified GPLs were separated into two fractions – polar and apolar GPLs – by scratching silica off a GPL-loaded TLC plate. For treatments, the lipid antigens were solubilized in distilled water containing 0.025% of Tween-80 (Sigma-Aldrich, St. Louis, MO, USA) using sonication.

### Macrophage infection and stimulation

BMDMs and RAW 264.7 cells were cultured in Dulbecco’s modified Eagle’s medium (DMEM, Sigma-Aldrich) supplemented with 10% of fetal bovine serum (FBS, Sigma-Aldrich). BMDMs were isolated from C57BL/6 mice as described elsewhere.^44^ BMDMs and RAW 264.7 cells were pretreated with GPL, *N*-acetyl-L-cysteine (NAC, Sigma-Aldrich), or cyclosporin A (Sigma-Aldrich) for 2 h, washed, and stimulated with apoptotic factors or infected with MAB at indicated MOIs. The cells were incubated with apoptotic inducers staurosporine (100 nM, Enzo Life Sciences, Farmingdale, NY, USA), H_2_O_2_ (0.5 mM, Sigma-Aldrich), TNF-*α* (20 ng/ml, eBioscience, San Diego, CA, USA) plus 2 *μ*g/ml cycloheximide (Enzo Life Sciences), or oligomycin (5 *μ*g/ml, Sigma-Aldrich) for 24 h. For quantification of colony-forming units (CFUs), the infected cells were lysed in distilled water and plated on 7H10 agar plates supplemented with OADC.

### Apoptosis analysis

The FITC Apoptosis Detection Kit I (BD) and a terminal deoxynucleotidyl transferase dUTP nick-end labeling (TUNEL) assay by means of the APO-BRDU Kit (Enzo Life Sciences) were used for apoptosis analysis. Caspase 3 and 9 activities were measured with Caspase 3 and 9 Fluorometric Assay Kits (Enzo Life Sciences). For confocal microscopy imaging, DEVD-FMK and LEHD-FMK reagents from the FAM-FlICA caspase detection kit (Immunochemistry Technologies, Bloomington, MN, USA) were used.

### Assessment of ROS production and ΔΨm

To quantify cellular ROS, the treated RAW 264.7 cells and BMDMs were stained with 10 *μ*M dichlorofluorescein diacetate (DCFDA, Sigma-Aldrich) and analyzed by FACS. Mitochondrial ROS were quantified by FACS using Mito-SOX staining (Life Technologies, Carlsbad, CA, USA). ΔΨm was determined by FACS using 10 nM dihexyloxacarbocyanine iodide (DiOC_6_, Sigma-Aldrich) staining, as described previously.^30^

### Subcellular fractionation and immunoblotting

Subcellular fractionation was performed using subcellular fractionation kits (Thermo Fisher Scientific, Waltham, MA, USA). Components of subcellular fractions or of the total cell lysate were separated by SDS-PAGE and then transferred onto a polyvinylidene difluoride (PVDF) membrane. For dot blotting, 2 *μ*l of cell fractions was directly dotted on the membrane. The blots were blocked with 10% skim milk in PBS and reacted with appropriate antibodies: anti-Bak, anti-Bax, anti-cytochrome *c*, anti-VDAC, anti-cyclophilin D, and anti-*α*-tubulin antibodies (Cell Signaling Technology, Danvers, MA, USA). Horseradish peroxidase-conjugated anti-mouse or anti-rabbit IgG antibodies (Calbiochem, San Diego, CA, USA) served as secondary antibodies. The anti-GPL antibody was raised in BALB/c mice immunized with GPL emulsified in Freund’s adjuvant (Sigma-Aldrich) in our laboratory. For immunoprecipitation, GPL-treated RAW 264.7 cells were disrupted in lysis buffer (50 mM Tris-Cl, 137 mM NaCl, 1 mM EDTA, 1% Triton X-100, 1 mM phenylmethylsulfonyl fluoride (PMSF), 10% glycerol, pH 8.0). The lysates were incubated with antibodies against cyclophilin D (Enzo Life Sciences) or GPL and protein A Dynabeads (Life Technologies). The final reactants were heated at 70 °C for 10 min and subjected to an immunoblot assay.

### Confocal microscopy

BMDMs infected with MAB or treated with GPL for 2 h were washed with PBS and DMEM containing antibiotics for elimination of MAB present in the medium. After 4 h, the cells were fixed in 4% paraformaldehyde and permeabilized with 0.1% Triton-X 100. The fixed cells were stained with Alexa 488- and Alexa 568-conjugated anti-mouse and anti-rabbit IgG antibodies and fluorometric dyes (DAPI, MitoTracker, Mito-SOX; Life Technologies).

### Bacterial spreading assay

RAW 264.7 cells were infected with RFP-expressing R-type MAB. After 2 h of infection, the infected cells were harvested and washed with PBS two times, then mixed with recipient RAW 264.7 cells stained with a cell tracker blue CMAC (Thermo Fisher Scientific).

### Statistical analysis

All the experiments were performed at least twice. All data are expressed as mean±S.D. or S.E.M., and statistical analyses were conducted in GraphPad Prism version 5 (GraphPad Software, San Diego, CA, USA). Comparisons were made by two-way ANOVA with Bonferroni’s *post hoc* test for multiple groups of samples, and one-way ANOVA with Tukey’s *post hoc* test for pairwise comparisons. Differences with *P-*values of <0.05 were considered significant.
