# Supplementary material for: Mycobacterium abscessus glycopeptidolipids inhibit macrophage apoptosis and bacterial spreading by targeting mitochondrial cyclophilin D
Source: Cell Death Dis. 2017 Aug 24;8(8):e3012–. doi: 10.1038/cddis.2017.420 (PMC5596598; doi:10.1038/cddis.2017.420)
Supplement: Supplementary Figures [file cddis2017420x1.ppt]

## Slide 1
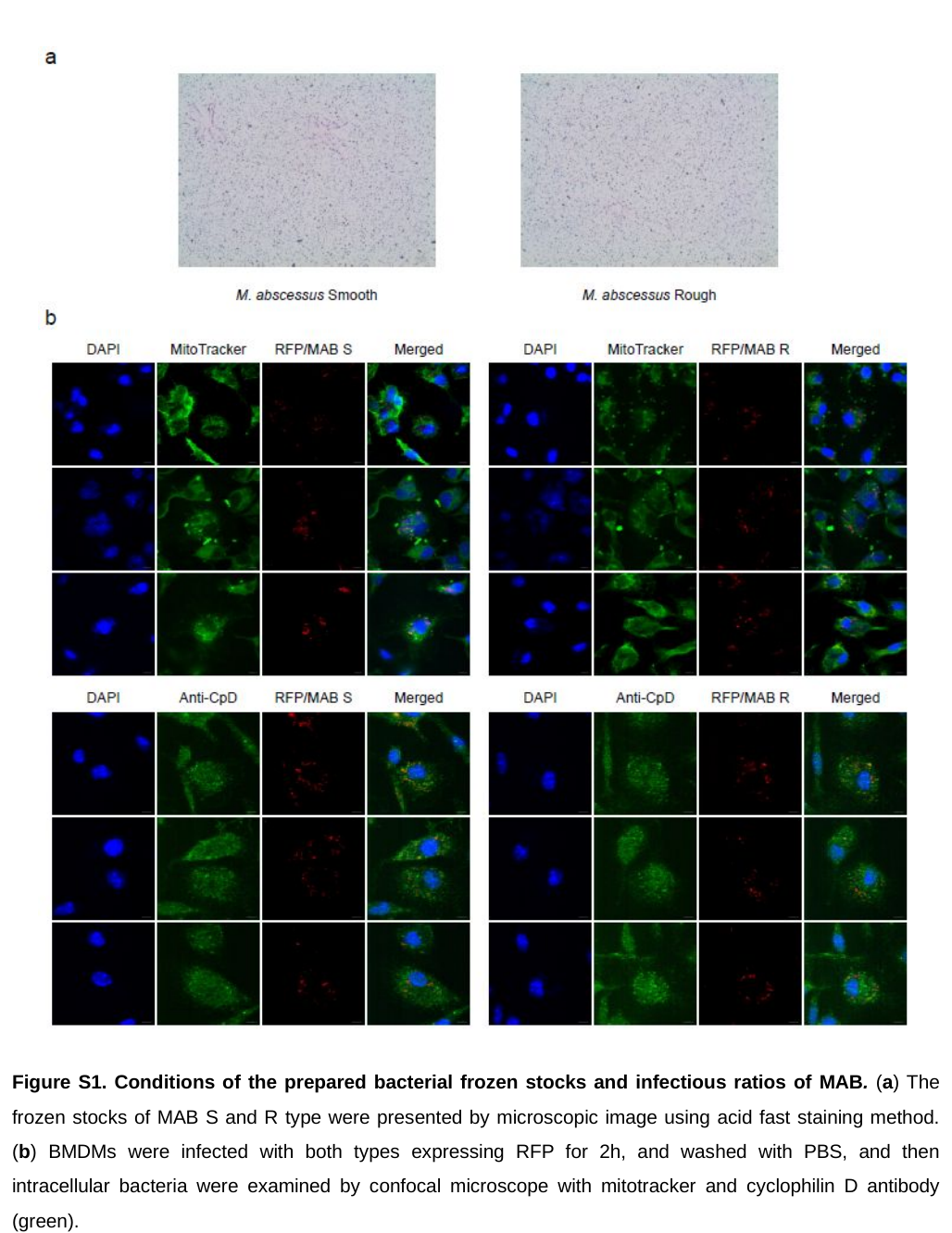

Figure S1. Conditions of the prepared bacterial frozen stocks and infectious ratios of MAB. (a) The frozen stocks of MAB S and R type were presented by microscopic image using acid fast staining method. (b) BMDMs were infected with both types expressing RFP for 2h, and washed with PBS, and then intracellular bacteria were examined by confocal microscope with mitotracker and cyclophilin D antibody (green).

## Slide 2
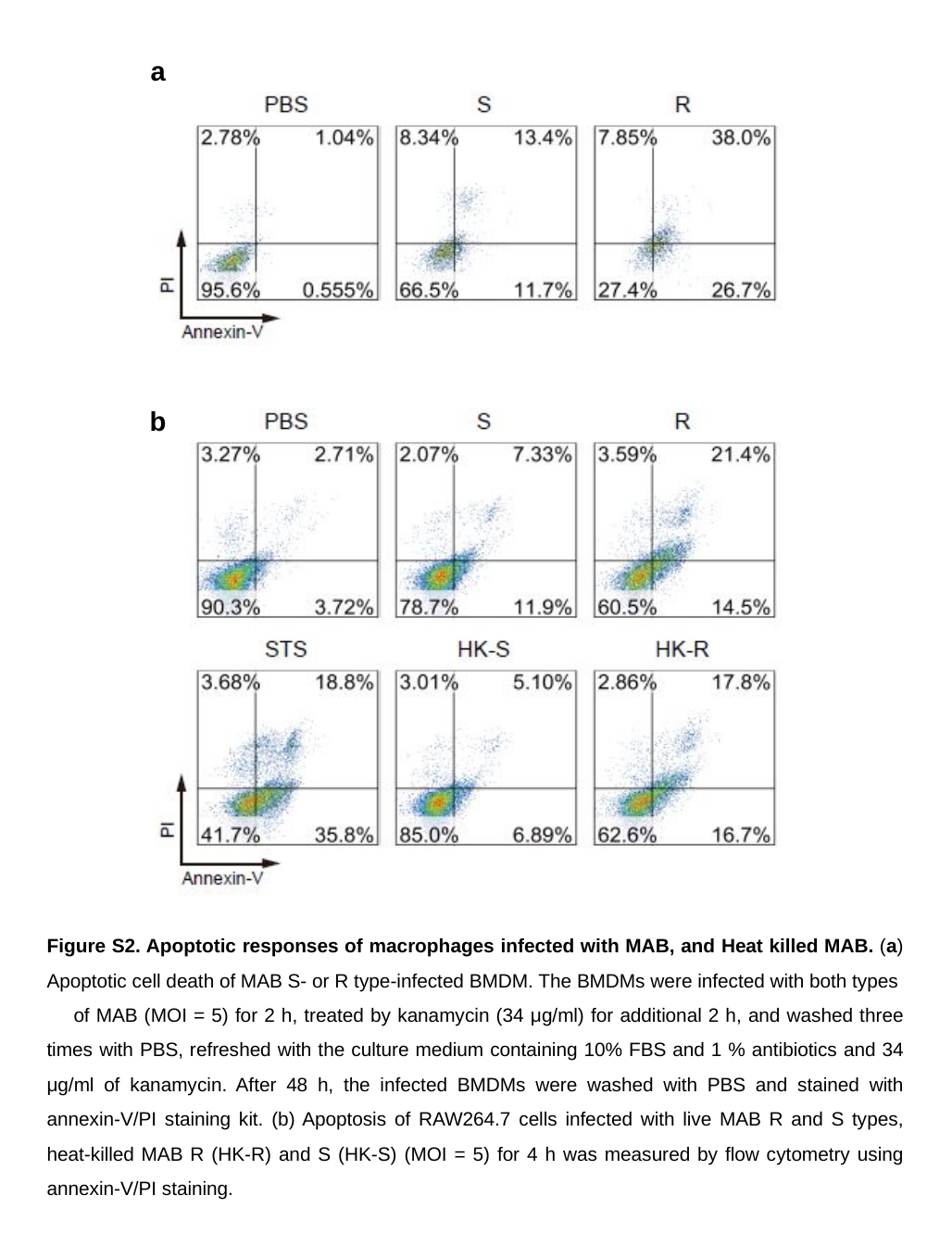

a
b
Figure S2. Apoptotic responses of macrophages infected with MAB, and Heat killed MAB. (a) Apoptotic cell death of MAB S- or R type-infected BMDM. The BMDMs were infected with both types of MAB (MOI = 5) for 2 h, treated by kanamycin (34 μg/ml) for additional 2 h, and washed three times with PBS, refreshed with the culture medium containing 10% FBS and 1 % antibiotics and 34 μg/ml of kanamycin. After 48 h, the infected BMDMs were washed with PBS and stained with annexin-V/PI staining kit. (b) Apoptosis of RAW264.7 cells infected with live MAB R and S types, heat-killed MAB R (HK-R) and S (HK-S) (MOI = 5) for 4 h was measured by flow cytometry using annexin-V/PI staining.

## Slide 3
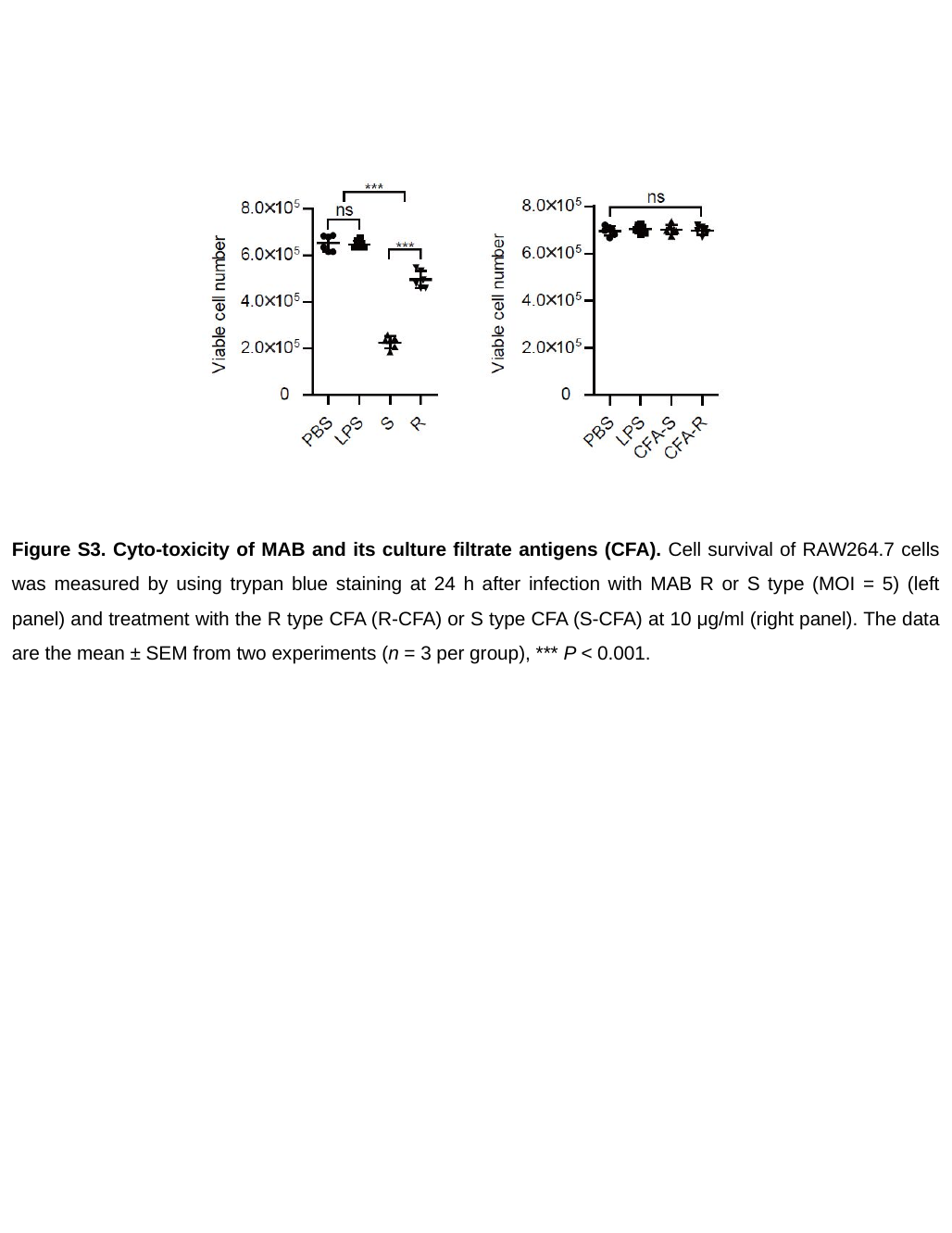

Figure S3. Cyto-toxicity of MAB and its culture filtrate antigens (CFA). Cell survival of RAW264.7 cells was measured by using trypan blue staining at 24 h after infection with MAB R or S type (MOI = 5) (left panel) and treatment with the R type CFA (R-CFA) or S type CFA (S-CFA) at 10 μg/ml (right panel). The data are the mean ± SEM from two experiments (n = 3 per group), *** P < 0.001.

## Slide 4
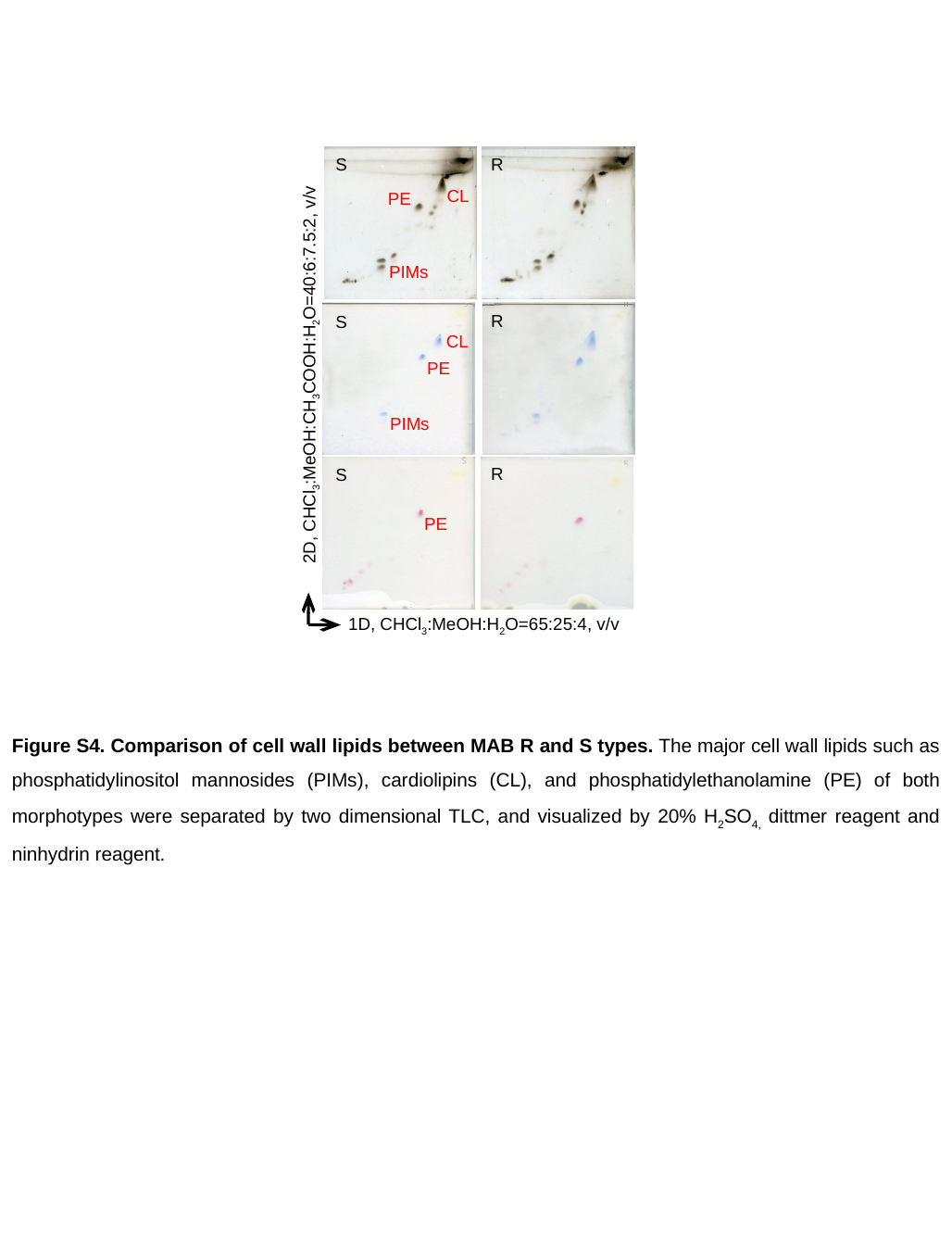

R
S
CL
PE
PIMs
R
S
CL
PE
2D, CHCl3:MeOH:CH3COOH:H2O=40:6:7.5:2, v/v
PIMs
R
S
PE
1D, CHCl3:MeOH:H2O=65:25:4, v/v
Figure S4. Comparison of cell wall lipids between MAB R and S types. The major cell wall lipids such as phosphatidylinositol mannosides (PIMs), cardiolipins (CL), and phosphatidylethanolamine (PE) of both morphotypes were separated by two dimensional TLC, and visualized by 20% H2SO4, dittmer reagent and ninhydrin reagent.

## Slide 5
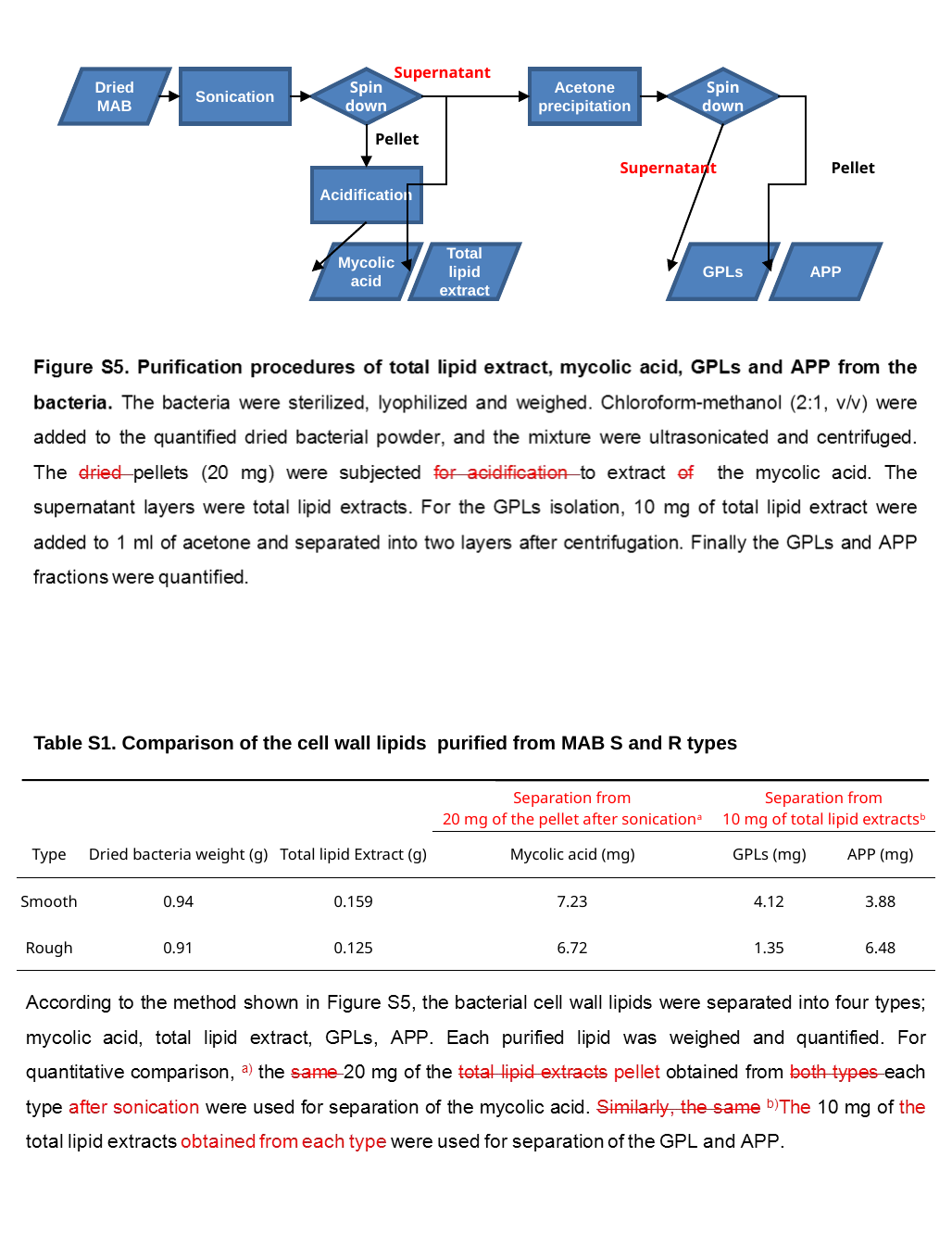

Supernatant
Dried MAB
Sonication
Spin down
Acetone precipitation
Spin down
Pellet
Supernatant
Pellet
Acidification
Mycolic acid
Total lipid extract
GPLs
APP
Table S1. Comparison of the cell wall lipids purified from MAB S and R types
| | | | Separation from 20 mg of the pellet after sonicationa | Separation from 10 mg of total lipid extractsb | |
| --- | --- | --- | --- | --- | --- |
| Type | Dried bacteria weight (g) | Total lipid Extract (g) | Mycolic acid (mg) | GPLs (mg) | APP (mg) |
| Smooth | 0.94 | 0.159 | 7.23 | 4.12 | 3.88 |
| Rough | 0.91 | 0.125 | 6.72 | 1.35 | 6.48 |

## Slide 6
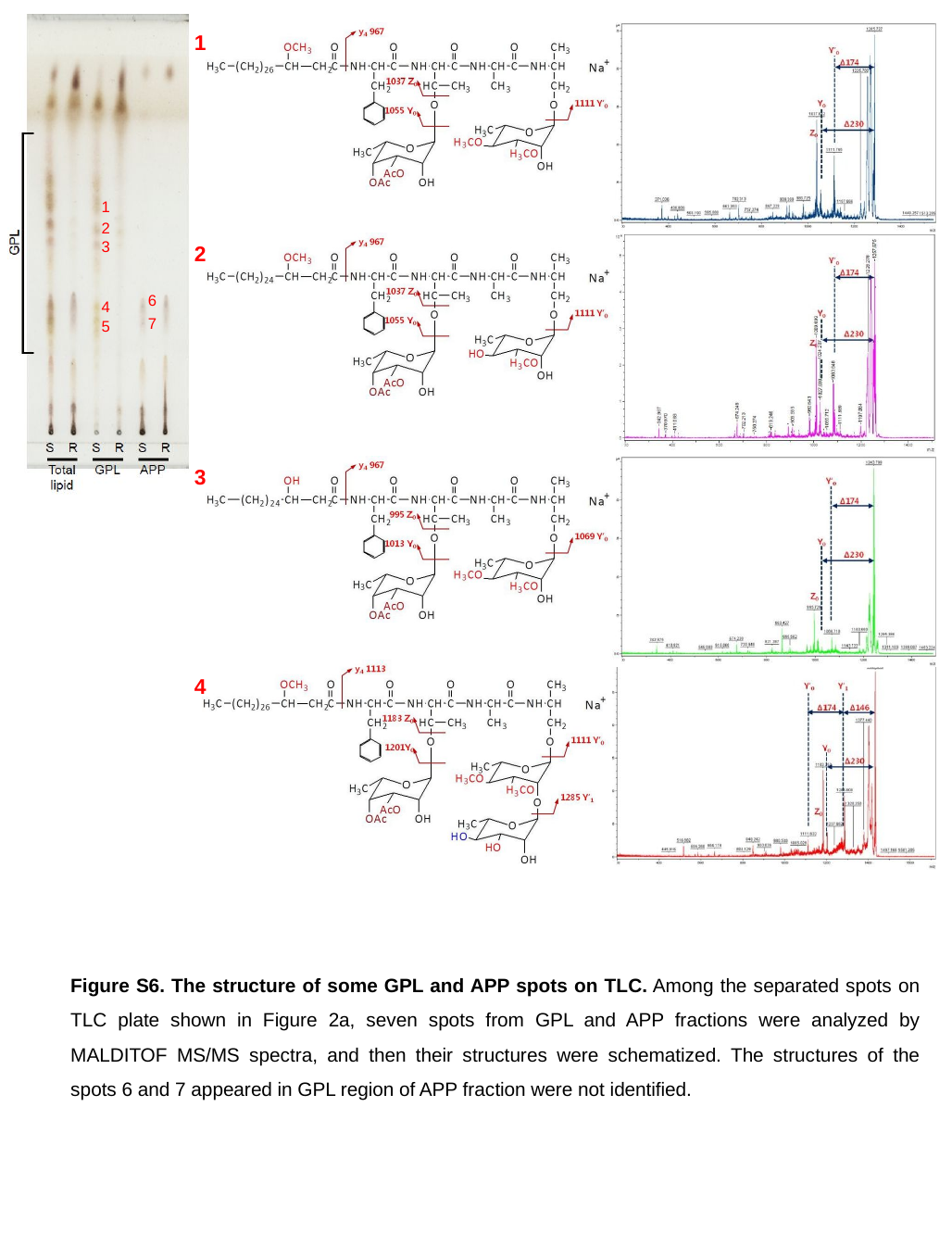

1
1
2
3
2
6
4
7
5
3
4
Figure S6. The structure of some GPL and APP spots on TLC. Among the separated spots on TLC plate shown in Figure 2a, seven spots from GPL and APP fractions were analyzed by MALDITOF MS/MS spectra, and then their structures were schematized. The structures of the spots 6 and 7 appeared in GPL region of APP fraction were not identified.

## Slide 7
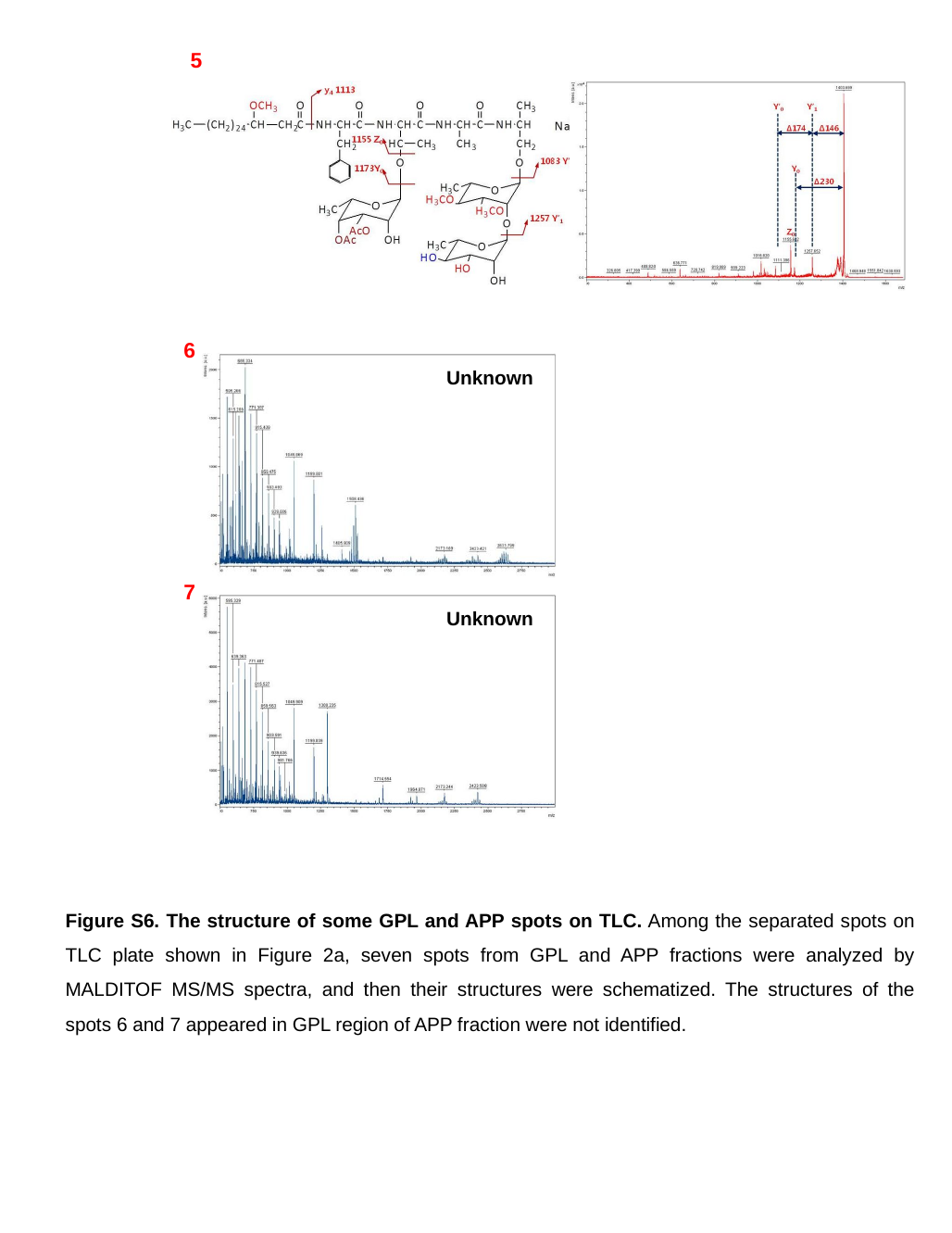

5
6
Unknown
7
Unknown
Figure S6. The structure of some GPL and APP spots on TLC. Among the separated spots on TLC plate shown in Figure 2a, seven spots from GPL and APP fractions were analyzed by MALDITOF MS/MS spectra, and then their structures were schematized. The structures of the spots 6 and 7 appeared in GPL region of APP fraction were not identified.

## Slide 8
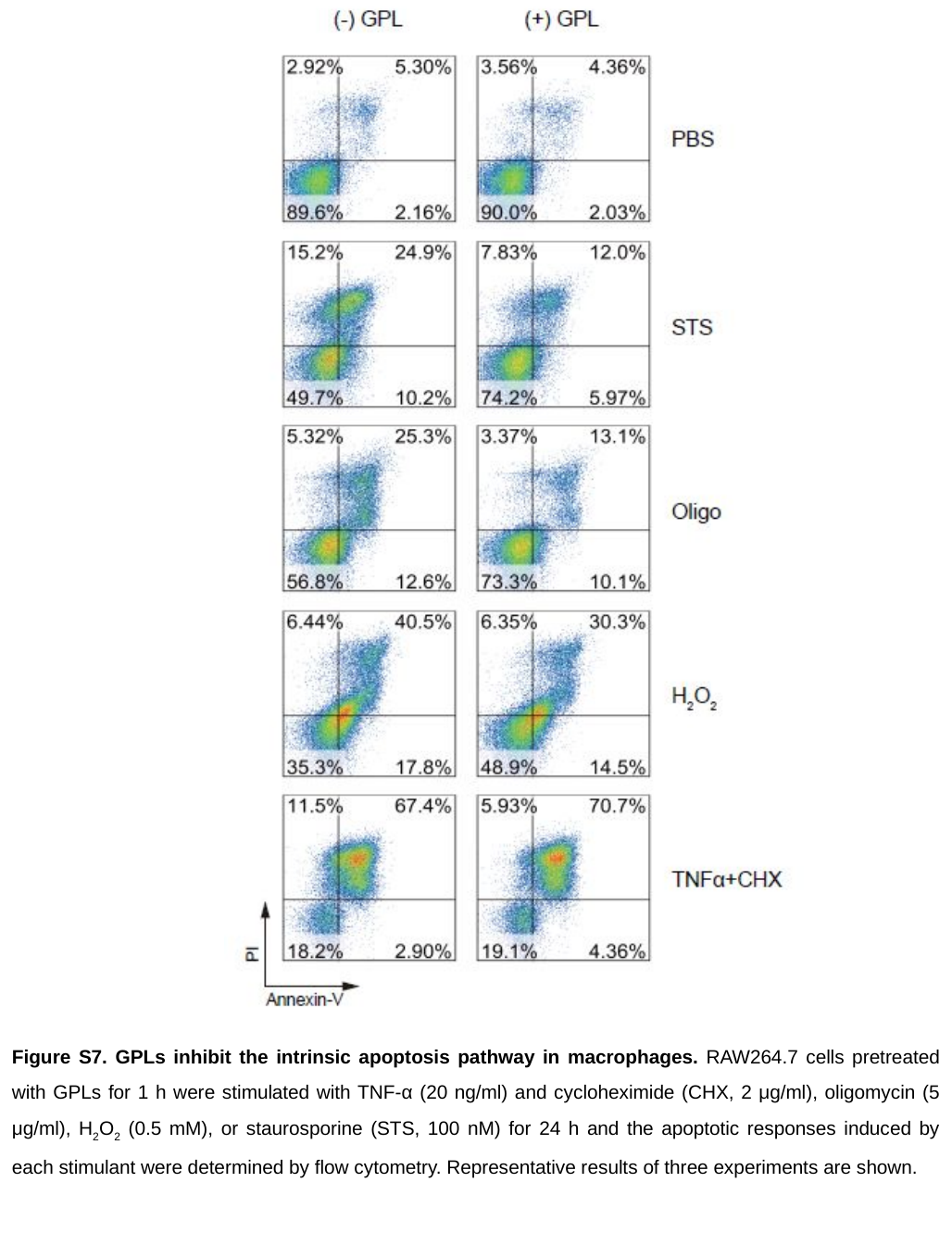

Figure S7. GPLs inhibit the intrinsic apoptosis pathway in macrophages. RAW264.7 cells pretreated with GPLs for 1 h were stimulated with TNF-α (20 ng/ml) and cycloheximide (CHX, 2 μg/ml), oligomycin (5 μg/ml), H2O2 (0.5 mM), or staurosporine (STS, 100 nM) for 24 h and the apoptotic responses induced by each stimulant were determined by flow cytometry. Representative results of three experiments are shown.

## Slide 9
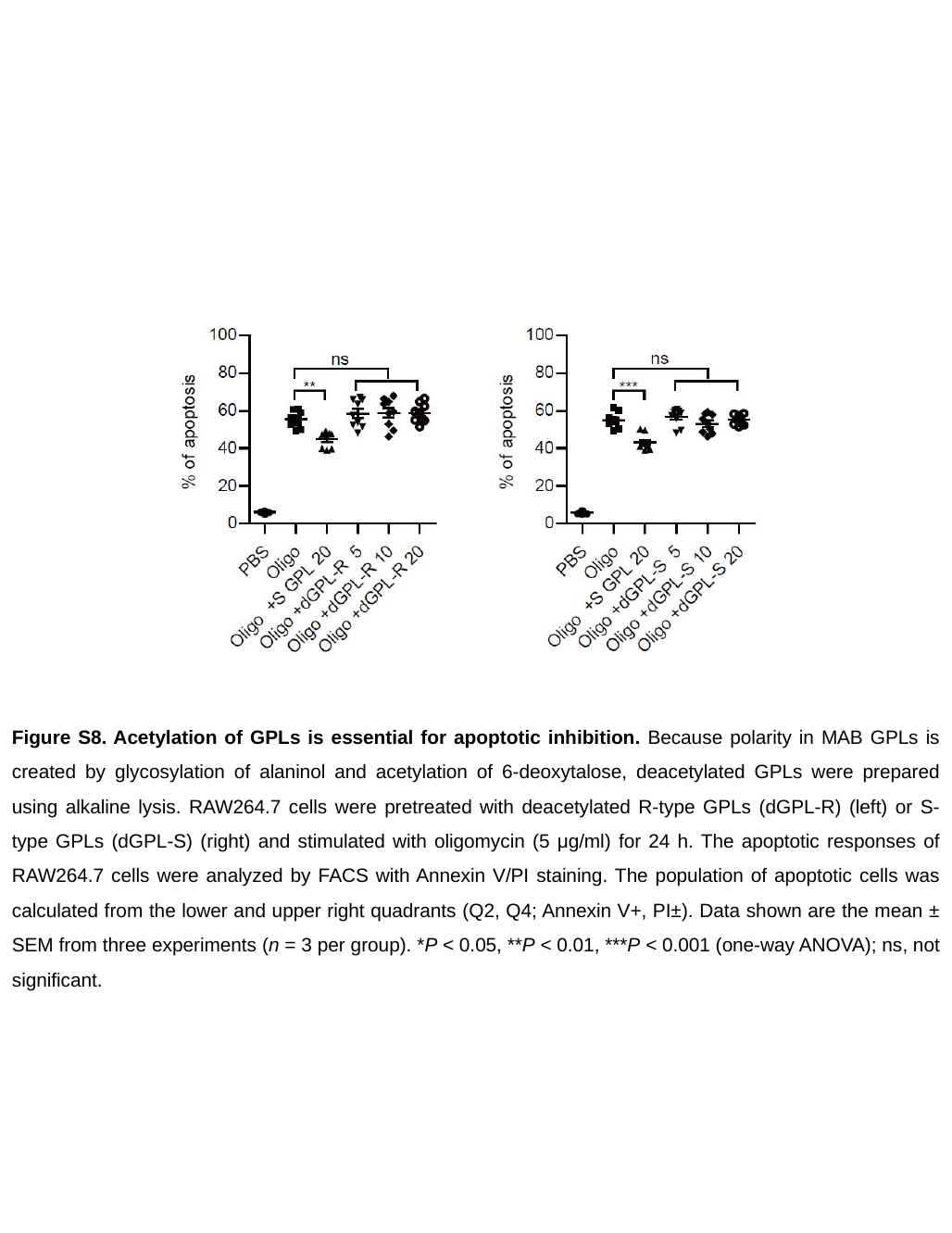

Figure S8. Acetylation of GPLs is essential for apoptotic inhibition. Because polarity in MAB GPLs is created by glycosylation of alaninol and acetylation of 6-deoxytalose, deacetylated GPLs were prepared using alkaline lysis. RAW264.7 cells were pretreated with deacetylated R-type GPLs (dGPL-R) (left) or S-type GPLs (dGPL-S) (right) and stimulated with oligomycin (5 μg/ml) for 24 h. The apoptotic responses of RAW264.7 cells were analyzed by FACS with Annexin V/PI staining. The population of apoptotic cells was calculated from the lower and upper right quadrants (Q2, Q4; Annexin V+, PI±). Data shown are the mean ± SEM from three experiments (n = 3 per group). *P < 0.05, **P < 0.01, ***P < 0.001 (one-way ANOVA); ns, not significant.
